# Adding Time Dimension to Photosynthesis Studies: A Path Toward Linking Biomass to Photosynthesis

**DOI:** 10.1111/ppl.70422

**Published:** 2025-07-31

**Authors:** Sofia A. Bengoa Luoni, Steven M. Driever

**Affiliations:** ^1^ Laboratory of Genetics Wageningen University & Research Wageningen the Netherlands; ^2^ Centre for Crop Systems Analysis Wageningen University & Research Wageningen the Netherlands

**Keywords:** biomass, daily electron integral, FluorCam, photosynthesis, projected leaf area

## Abstract

Biomass is a key indicator of growth rate, resource use efficiency, and overall plant performance. Biomass is ultimately driven by photosynthesis, as it reflects the plant's ability to convert light energy into chemical energy and build structural and storage tissues over time. In recent years, high‐throughput photosynthesis phenotyping methods have been developed using pulse‐amplitude modulated chlorophyll fluorescence. These methods allow fast whole‐plant measurements that can be taken daily and multiple times per day, offering the opportunity to capture variation in photosynthesis over time. In this study, we introduce a novel method to estimate the above‐ground dry biomass of three plant species by utilizing the daily electron integral (DEI) as a correction factor for plant‐projected areas. This new approach considers whole plant measurements of the operating efficiency of PSII, as well as time variation within the day and across the experiment. Our findings demonstrate a strong linear correlation between this new index and biomass under two light conditions, evidenced by an *R* ^2^ value of 0.98.

## 1 Introduction

Biomass is a key indicator of growth rate, resource use efficiency, and overall plant performance (Hunt 1990). It reflects how well a plant converts inputs (light, water, nutrients) into tissue. Biomass is often used as a selection trait in breeding programs to reflect tolerance to stress or nutrient use efficiency (Chaurasia et al. 2020; Cafaro La Menza et al. 2022).

Destructive methods remain the standard for accurately measuring plant biomass at a given time, but they are time‐consuming and space‐intensive, as the number of plants required to assess biomass over time increases rapidly. Non‐destructive biomass estimations mostly rely on imaging‐derived projected leaf area (PLA) (Schiphorst et al. 2023). Although PLA is faster to assess, it often shows a weak linear correlation (*R* ~ 0.8) with above‐ground dry biomass, and it is not comparable across experiments (Weraduwage et al. 2015; Yang et al. 2017). This discrepancy arises not only from differential allocation to stems or reproductive organs but also from variations in leaf mass per area (LMA) among species, genotypes, and growing conditions.

There is a direct relation between biomass and photosynthesis. Biomass is driven by photosynthetic carbon assimilation, and new methods have been developed to measure photosynthetic parameters in a fast and accurate way. High‐throughput photosynthesis phenotyping methods employ a pulse‐amplitude modulated chlorophyll fluorescence technique to calculate the operating efficiency of photosystem II (PSII) or Φ_PSII_. In turn, Φ_PSII_ together with a known light intensity and leaf absorptance, allows for the calculation of an electron transport rate (ETR) through PSII. Both these parameters (Φ_PSII_, ETR) are correlated with CO_2_ assimilation rates (Genty et al. 1989). The advantage of Φ_PSII_ measurement is that it allows for rapid data collection and can be averaged over the entire plant if whole plant images are taken.

Even more, this system can capture variation of Φ_PSII_ across time. Photosynthesis varies across each leaf in the canopy and throughout the different stages of leaf development. Additionally, diurnal changes influenced by the circadian clock affect photosynthesis, typically resulting in higher rates in the morning compared to the afternoon (Haydon and Webb 2016).

In this study, we evaluate the correlation between photosynthesis and above‐ground dry biomass using the cumulative daily electron integral (∑aDEI). DEI was obtained by modeling ETR using a polynomial function per day to incorporate the variance of photosynthetic operating efficiency within and across the day. We assessed three related species that exhibit significant differences in LMA under two light conditions.

## 2 Materials and Methods

### 2.1 Experimental Design

Two experiments were conducted under controlled environmental conditions, one under low light (LL) at 250 μmol m^−2^ s^−1^ and the other under high light (HL) at 1800 μmol m^−2^ s^−1^. Three plant species were analyzed: *Brassica rapa* (accession R‐o‐18), *Brassica nigra* (accession DG1), and *Hirschfeldia incana* (accession Nijmegen). A total of 20 plants per species were used in the LL experiment and 45 plants per species in the HL experiment.

Plants were germinated under a light intensity of either 250 or 500 μmol m^−2^ s^−1^, corresponding to the low light (LL) and high light (HL) conditions, respectively, and allowed to grow for 4 days. Subsequently, seedlings showing two healthy cotyledons were transplanted into the high‐throughput photosynthesis phenotyping chamber (www.npec.nl).

The plants were grown in a high‐throughput photosynthesis phenotyping chamber where the conditions were maintained at a CO₂ concentration of 440 ppm, a relative humidity of 70%, and a day/night temperature of 22°C/18°C. The plants were subjected to a photoperiod of 12 h, with a light intensity of 250 or 1800 μmol m^−2^ s^−1^ PAR (LL and HL conditions, respectively). The light intensity gradually increased or decreased over the course of 1 h at the start and the end of the photoperiod each day.

A third experiment was conducted under the environmental conditions described above, using a subdivided growth chamber to simultaneously generate both light treatments. In this experiment, plants were used exclusively to calculate leaf mass per area (LMA).

### 2.2 LMA

A total of five plants per species and per light condition were harvested at 24 and 22 DAS (LL, HL, respectively) to determine the LMA, expressed in g m^−2^ for each leaf of each plant. Leaves were separated from the plants, and RGB images were taken using a Nikon camera. Leaf areas were calculated using an RGB image‐based Python pipeline (Gehan et al. 2017). Leaf tissues were then dried in an oven at a constant temperature of 65°C until a stable dry weight was reached. The dry mass of each leaf was measured using a precision balance (see Supporting Information 1).

### 2.3 Dry Biomass Measurement

A total of five plants per species were harvested at 14, 16, and 21 DAS for the LL experiment, and 10 plants per species at 15, 18, 22, and 28 DAS for the HL experiment. The dry above‐ground biomass was measured as follows: first, each plant shoot was carefully separated from the rockwool block, ensuring that roots were not included. Shoots were then dried in an oven at a constant temperature of 65°C until a stable dry weight was reached. The dry weight of each shoot was recorded using a precision balance (see Supporting Information 2).

### 2.4 Phenotyping

The high‐throughput photosynthesis phenotyping chamber NPEC‐module4‐G8 is equipped with a motorized XYZ gantry system capable of reaching each plant within a designated area (www.npec.nl). The gantry includes a camera system featuring a FluorCam (Photon Systems Instruments), which specializes in capturing photosynthetic measurements.

Φ_PSII_ measurements (Equation 1) were conducted daily as previously described (IntelligentMasking, Bengoa Luoni et al. 2024; Murchie and Lawson 2013) beginning at 30 min after exposure to 250 or 1800 μmol m^−2^ s^−1^ PAR and subsequently at noon and in the afternoon. Projected leaf area (PLA) measurements were first calculated in pixels from the morning *F*_m_′ images and then converted to square meters based on the camera's field of view.

(1) $$\Phi_{\text{PSII}}=\left(F_{m}-F_{s}\right)/F_{m}$$

where *F*_s_ is steady‐state fluorescence (light‐adapted) and *F*_m_ is maximal fluorescence in the light‐adapted state.

### 2.5 DEI (mol Electrons m^−2^)

Each plant was analyzed individually, initially with daily data. For each day, the electron transport rate (ETR) corresponding to each Φ_PSII_ measurement was computed (Equation 2).

(2) $$\mathrm{ETR}=\Phi_{\text{PSII}}\times I\times \alpha \times \mathrm{PSI}/\text{PSII}$$

Incident light intensity (*I*) is measured in μmol m^−2^ s^−1^. The absorptance of light (*α*), which represents the fraction of incoming light absorbed by the leaf, is assumed to be 0.84. The ratio of Photosystem II to Photosystem I (PSII/PSI) is assumed to be 0.5.

To obtain the DEI, Daily Electron Integral, the ETR was subsequently parameterized as a temporal function (in seconds) starting from the initiation of each Φ_PSII_ measurement, employing a quadratic polynomial function. The function was integrated between the morning and afternoon measurements (Supporting Information 3, 4).

### 2.6 aDEI (μmol Electrons)

The projected leaf area (PLA) values were initially obtained as pixel counts from *F*_m_′ images. These values were then converted to square meters (m^2^) based on the camera's field of view, which covers an area of 20 × 20 cm and corresponds to an image resolution of 1024 × 1360 pixels.

The absolute value of the daily electron integral (aDEI) was calculated by multiplying each ETR data point by the PLA (m^2^). Following a similar procedure, each ETR × PLA value was parameterized as a function of time (s). Subsequently, a polynomial function was fitted and integrated over each day.

### 2.7 ∑aDEI (μmol Electrons)

Finally, the daily values of aDEI were summed over the light cycle from the day of the first measurement of the experiment until the harvest date (see Supporting Information 2).

## 3 Results

**FIGURE 1:**
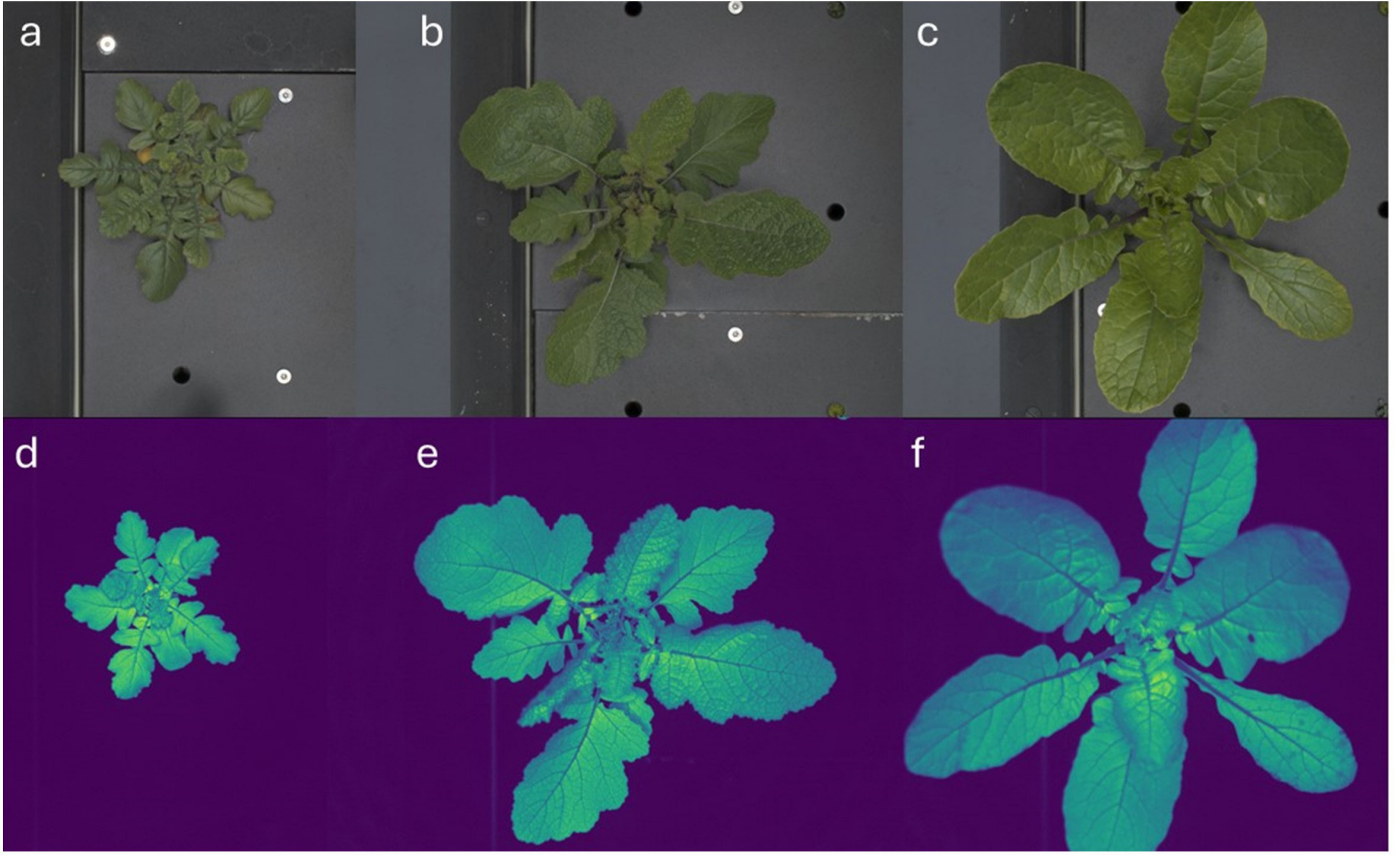
Top‐view image examples of RGB pictures (a–c) and chlorophyll fluorescence pictures (d–f). Three species belonging to the Brassicaceae family were studied: *H. incana* (a, d), *B. nigra* (b, e), and *B. rapa* (c, f). The experiment was considered finished when the larger species ( *B. rapa* ) could no longer be seen in the field of view of the fluorescence camera (f).

To evaluate the daily electron integral (DEI) as an indicator of biomass, two experiments were conducted under light intensities of 250 μmol m^−2^ s^−1^ (low light, LL) and 1800 μmol m^−2^ s^−1^ (high light, HL). Plants were grown in the high‐throughput phenotyping facility at NPEC (www.npec.nl). The PSI XYZ system (Photon Systems Instruments) was used to capture top‐view RGB and chlorophyll fluorescence images of three plant species, *Hirschfeldia incana* , *Brassica rapa* , and *Brassica nigra* , over time (Figure 1). Plants were harvested at various time points to measure above‐ground biomass.

These images were subsequently processed to calculate Φ_PSII_ using a custom analysis method based on raw fluorescence data (IntelligentMasking, (Bengoa Luoni et al. 2024)). To capture the dependency of photosynthesis on the circadian clock, three measurements were taken per day—morning, noon, and afternoon. This method allowed for rapid and accurate phenotyping across a large number of plants, enhancing the precision of our analysis (Figure 1).

**FIGURE 2:**
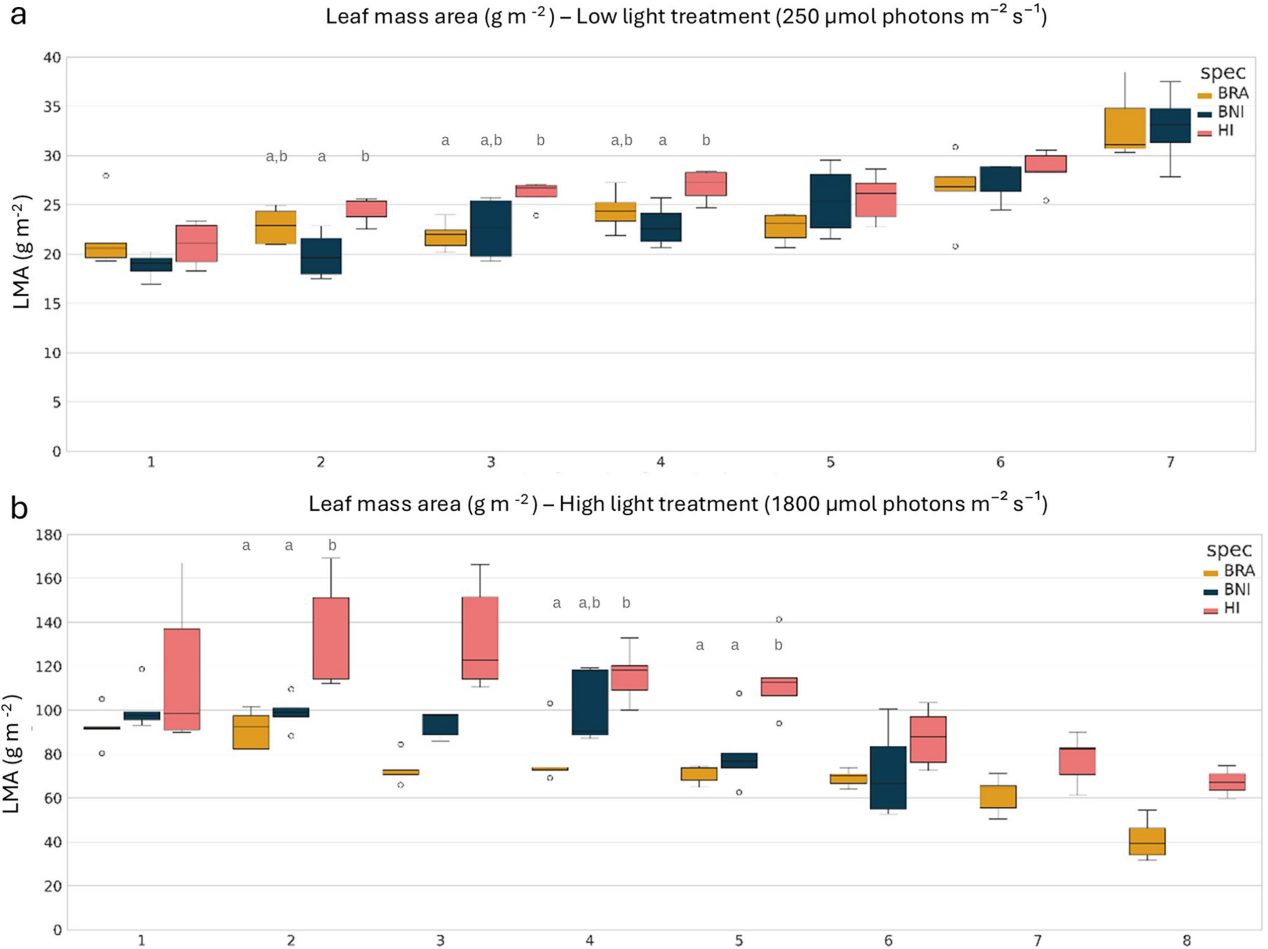
Leaf mass per area (LMA, g m^−2^) of individual leaves under (a) low light (250.0 μmol m^−2^ s^−1^) or (b) high light treatment (1800.0 μmol m^−2^ s^−1^). Each boxplot represents the LMA distribution (*y*‐axe) for a given leaf number (ordered by their appearance, *x*‐axe) across five plants per species. Species are indicated by color: *Brassica rapa* (BRA, yellow), *Brassica nigra* (BNI, dark blue), and *Hirschfeldia incana* (HI, pink). An ANOVA followed by Tukey's HSD test was performed to compare LMA values between species for each leaf position. Letters above the bars indicate statistically significant differences (*p* < 0.05).

LMA, a proxy for leaf thickness, was calculated for each leaf of five plants per species at 24 DAS (LL experiment) or 22 DAS (HL experiment). Figure 2 shows the LMA of each leaf, ordered by appearance. At the time of harvest, *B. rapa* and *H. incana* had more leaves than *B. nigra* in both light treatments.

As expected, plants grown under HL displayed significantly higher LMA values (140 g m^−2^) than those grown under LL (35 g m^−2^). When comparing species within light treatments, LMA of *H. incana* was significantly higher than LMA of the other species, especially under HL (Leaf 2 and Leaf 5).

PLA, projected leaf area, is one of the easiest non‐destructive methods to estimate biomass and is commonly used as a routine method in phenotyping facilities (Weraduwage et al. 2015). The PLA values were calculated for each experiment using *F*_m_′ top‐view pictures of the three species, and these values were correlated with above‐ground biomass using a linear regression. At the time points analyzed, we observed clear differences in plant size, with *H. incana* being the smallest of the group.

**FIGURE 3:**
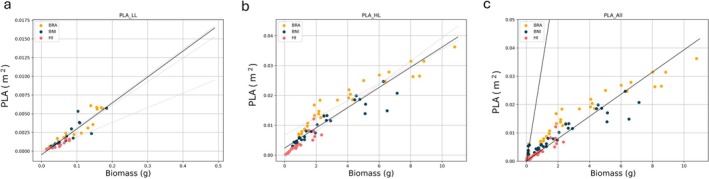
Scatter plots depicting above‐ground dry biomass (in grams) with PLA (in m^2^) for three different species (BRA: *B. rapa* , yellow; BNI: *B. nigra*, blue; HI: *H. incana* , pink dots). (a) Low light (250 μmol m^−2^ s^−1^) or (b) high light treatment (1800 μmol m^−2^ s^−1^), (c) both experiment analyzed together. Overlaid linear regression lines (solid lines) are shown for the three different species in light gray or analyzed in conjunction in black.

Figure 3 shows that there was a strong correlation in both experiments for each species (corresponding *R* ^2^ values are given in Table 1). However, differences in the correlation persist when analyzing individual species, under the same light treatment—for example, the slope for *H. incana* under low light (LL) is 0.0195 m^2^ g^−1^, compared to 0.0348 m^2^ g^−1^ for *B. rapa* . These differences suggest that the parameter correlates better with plants belonging to the same species. However, this result may be influenced by plant size and by the range over which the regression is analyzed.

**TABLE 1:** Table showing the relationship between biomass (g) and two predictors—∑aDEI (μmol electrons) and PLA (m^2^)—across different species (HI: *H. incana* , BRA: *B. rapa* , BNI: *B. nigra* ).

Treatment	Species	*y*	Slope	Intercept	*R* ^2^
LL	HI, BRA, BNI	PLA (m^2^)	0.034591	−0.00048	0.838
LL	HI	PLA (m^2^)	0.019545	−7.6E‐05	0.905
LL	BRA	PLA (m^2^)	0.034804	−0.00034	0.836
LL	BNI	PLA (m^2^)	0.031547	−0.00018	0.695
HL	HI, BRA, BNI	PLA (m^2^)	0.003381	0.002378	0.882
HL	HI	PLA (m^2^)	0.003919	0.000128	0.802
HL	BRA	PLA (m^2^)	0.002866	0.006768	0.91
HL	BNI	PLA (m^2^)	0.002862	0.002848	0.874
LL, HL	HI, BRA, BNI	PLA (m^2^)	0.003426	0.002175	0.896
LL	HI, BRA, BNI	SaDEI (μmols e)	219911.1	−5819.7	0.78
LL	HI	SaDEI (μmols e)	134953.8	−1867.1	0.917
LL	BRA	SaDEI (μmols e)	244628.3	−9297.5	0.75
LL	BNI	SaDEI (μmols e)	230,124	−6209	0.714
HL	HI, BRA, BNI	SaDEI (μmols e)	270844.6	−48751.5	0.982
HL	HI	SaDEI (μmols e)	205,251	−1755.2	0.956
HL	BRA	SaDEI (μmols e)	275159.4	−41197.2	0.979
HL	BNI	SaDEI (μmols e)	258603.3	−38417.6	0.979
LL, HL	HI, BRA, BNI	SaDEI (μmols e)	266971.3	−29883.9	0.985

*Note:* Each row represents a linear regression analysis performed on a specific dataset, as indicated in the “Treatment” and “Species” columns. Additional columns display the regression coefficients (slope and intercept) and the coefficient of determination (*R* ^2^). All regressions were performed using the Python package Scikit‐learn.

The differences in the slopes of the linear regression between experiments are even more pronounced (LL: 0.034591 m^2^ g^−1^; HL: 0.003381 m^2^ g^−1^), indicating that it is not possible to extrapolate or compare between experiments where the LMA values vary drastically.

**FIGURE 4:**
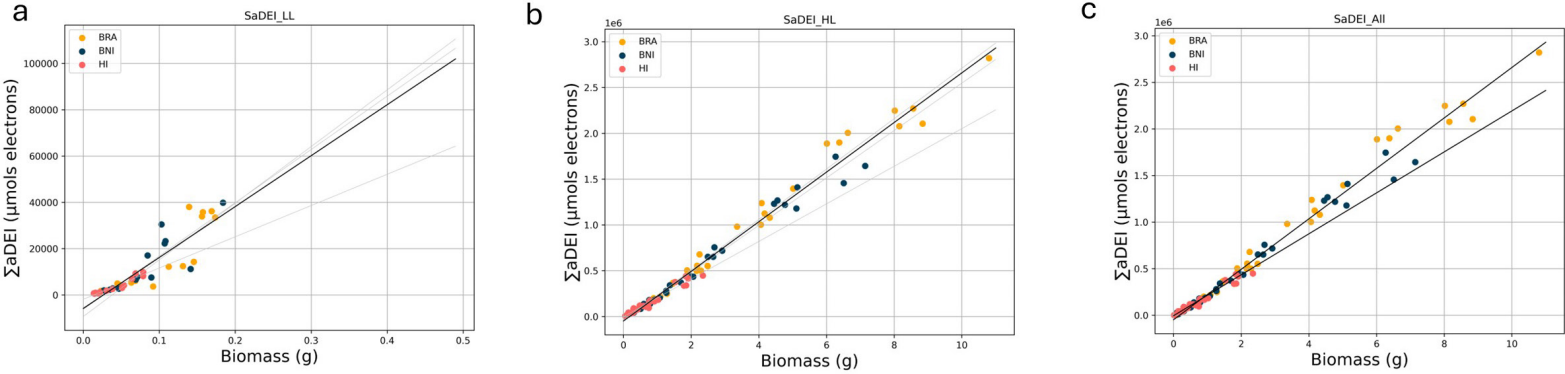
Scatter plots depicting above‐ground dry biomass (in grams) with ΣaDEI (in μmol e^−^) for three different species (BRA: *B. rapa* , yellow; BNI: *B. nigra*, blue; HI: *H. incana* , pink dots). (a) Low light (250 μmol m^−2^ s^−1^), (b) high light treatment (1800 μmol m^−2^ s^−1^), (c) both experiment analyzed together. Overlaid linear regression lines (solid lines) are shown for the three different species in light gray or analyzed in conjunction in black.

The absolute accumulated values of DEI (∑aDEI) and above‐ground dry biomass were analyzed through linear regression in both experiments individually and combined (Figure 4, Table 1). When comparing all species, we observed that the dispersion of the data in the low light (LL) experiment resulted in a lower *R* ^2^ for ∑aDEI (0.78) compared to PLA (0.83). However, in the high light (HL) experiment, the *R* ^2^ for ∑aDEI (0.98) was higher than that for PLA (0.88).

Interestingly, ∑aDEI showed consistent regression slopes across both experiments—slope 219,911 for LL and slope 270,844 for HL—suggesting that it may serve as a reliable indicator of biomass across different light conditions.

To evaluate which predictor—projected leaf area (PLA) or accumulated daily electron integral (∑aDEI)—better explains the variation in above‐ground dry biomass across species, we compared the Akaike Information Criterion (AIC) model performance. Given the substantial difference in scale between PLA (ranging from 0.0002137 to 0.03623 m^2^) and ∑aDEI (ranging from 541 to 2,820,365 μmol e), both variables were standardized using *z*‐score normalization prior to model fitting.

Model comparison based on AIC values revealed that PLA was a better predictor of biomass in the low light (LL) experiment (AIC: PLA = −318.46 vs. ∑aDEI = −306.00), whereas ∑aDEI performed substantially better under high light (HL) conditions (AIC: ∑aDEI = −194.91 vs. PLA = −29.91). When data from both experiments were analyzed together, ∑aDEI again showed a superior model fit (AIC = −327.58) compared to PLA (AIC = −77.94), suggesting that ∑aDEI may serve as a more robust predictor of biomass across varying light environments.

## 4 Discussion

The cumulative daily electron integral (∑aDEI) allowed direct correlation of above‐ground dry biomass with photosynthesis in a high‐throughput facility, using whole plant measurements of operating efficiency of PSII (Φ_PSII_). This is a novel, non‐destructive estimation of biomass, but also directly links to leaf and plant photosynthesis over time. Moreover, the same numerical relation can be implemented for different species and light conditions, being a useful tool to compare among genotypes and experiments.

The findings suggest that relying solely on PLA to estimate biomass may not always be reliable due to plants' adaptive responses, such as adjusting LMA, for example, in response to varying climatic conditions (Wright et al. 2004). Additionally, LMA varies significantly across different species and genotypes, complicating the direct relationship between leaf area and biomass. Correcting PLA with ETR has the advantage of considering the light conditions of the experiment.

On the other hand, relying solely on Φ_PSII_ measurements has been shown not to correlate well with biomass (Keller et al. 2024). It is well established that Φ_PSII_ decreases under high light conditions as a mechanism to regulate the amount of energy directed toward photochemistry. This trend was observed in our experiment, where the average Φ_PSII_ was 0.64 under low light (LL) and 0.23 under high light (HL), resulting in average ETR values of 67.9 and 174.9, respectively. A similar study linked the response of Φ_PSII_ to light intensities (ResponseG:PPFR) with above‐ground biomass in greenhouse‐grown plants and with grain yield (ton/ha) in field‐grown climbing bean genotypes (Keller et al. 2024).

In the aforementioned report, above‐ground biomass showed a correlation coefficient (*R*) ranging from 0.35 to 0.36 for the two analyzed greenhouse plots. For plants with a flat rosette‐like morphology, as is the case in our study, PLA alone already reached an *R* value of 0.89, and around 0.8 was reported in other studies (e.g., Yang et al. 2017). Therefore, the ResponseG:PPFR parameter does not offer an improvement in biomass prediction for such morphologies. However, this approach could be useful in studies involving species without a rosette‐type growth form, and it would be particularly interesting to compare it with the ∑aDEI metric approach presented in this report.

In this study, we performed two experiments under contrasting light regimes: one under LL and the HL conditions. Both experiments used a square light pattern with constant light intensity throughout the photoperiod (12 h). The LL treatment had a daily light integral (DLI) of approximately 10.8 mol m^−2^ day^−1^, while the HL treatment reached a DLI of 77.76 mol m^−2^ day^−1^. When polynomial functions were fitted to the ETR data of individual plants across the day, distinct temporal trends were observed. For example, under HL conditions on 2024‐05‐28, some individuals of *Brassica rapa* (BRA) showed a decline in ETR at noon and during afternoon measurements. However, the three species studied displayed relatively stable ETR values throughout the day (Supporting Information 4).

The duration of the experiments differed between light treatments—21 days for LL and 28 days for HL. As a result, plants reached larger sizes under HL, providing a broader range of phenotypic variation. This is clearly observed when analyzing LL and HL PLA together. While the walk‐in chamber setup used in this study ensured controlled conditions and consistent measurements, it also imposed limitations on plant size and growth duration. Although the PLA analysis across LL and HL conditions yields a relatively high *R* ^2^ (0.89), the data suggest that this correlation would weaken if larger plants were included under LL conditions. This is due to a clear divergence in the PLA slope between LL and HL, indicating distinct growth dynamics.

Extending the experimental period to include later developmental stages, such as stem elongation and flowering, and accommodating larger plants would be highly beneficial. Such extensions could be achieved using alternative platforms, such as automated or robotized greenhouses, which offer greater flexibility in plant size, growth conditions, and experimental duration.

These results encourage further investigation of ∑aDEI and their correlation with biomass in other species and under different environmental conditions, as well as refinement of the regression with measurements of absorptivity and species‐specific PSI/PSII ratios for a more precise calculation of ETR values. Moreover, since LMA variation is also found between leaves of the same species, a prior segmentation of each leaf and precise calculation of ETR for each leaf, including measured leaf absorptance, would be desirable.

## Author Contributions

S.A.B.L. designed and performed the experiments, conducted data analysis, wrote open‐source codes, and executed routines. S.A.B.L. conceived and revised the manuscript. S.M.D. addressed physiological aspects of photosynthesis and revised the manuscript. All authors have read and agreed to the published version of the manuscript.

## Supporting information


Data S1.

Data S2.

Data S3.

Data S3.

## Data Availability

All raw data obtained from the FluoroCam system (~500 GB) are available upon request. Please contact the corresponding author to arrange access.
