# Supplementary material for: Adding Time Dimension to Photosynthesis Studies: A Path Toward Linking Biomass to Photosynthesis
Source: Physiol Plant. 2025 Jul 31;177(4):e70422. doi: 10.1111/ppl.70422 (PMC12314342; doi:10.1111/ppl.70422)
Supplement: Supplementary file 4 — Data S3. [file PPL-177-e70422-s002.pptx]

## Slide 1
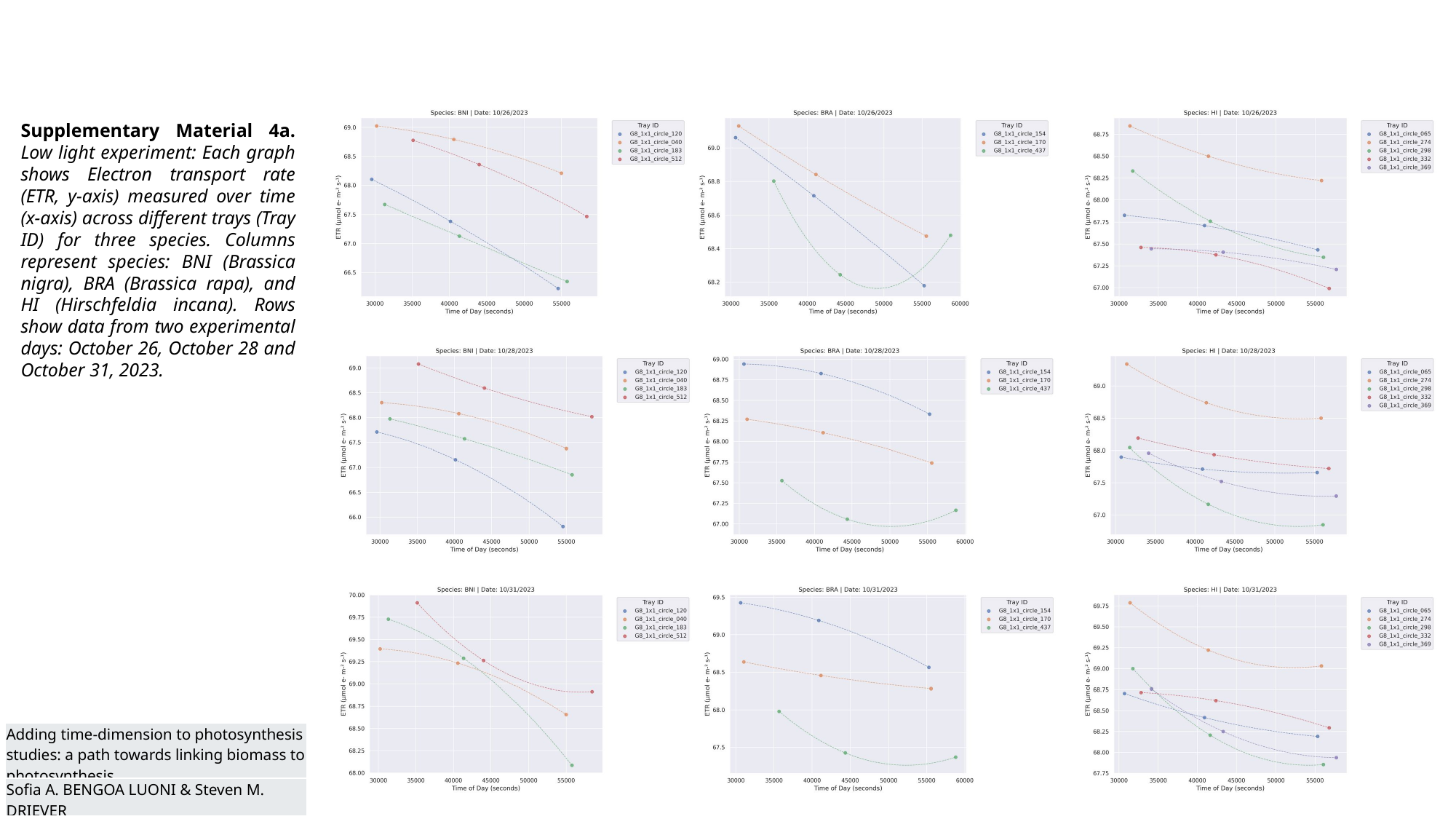

Supplementary Material 4a. Low light experiment: Each graph shows Electron transport rate (ETR, y-axis) measured over time (x-axis) across different trays (Tray ID) for three species. Columns represent species: BNI (Brassica nigra), BRA (Brassica rapa), and HI (Hirschfeldia incana). Rows show data from two experimental days: October 26, October 28 and October 31, 2023.
| Adding time-dimension to photosynthesis studies: a path towards linking biomass to photosynthesis |
| --- |
| Sofia A. BENGOA LUONI & Steven M. DRIEVER |

## Slide 2
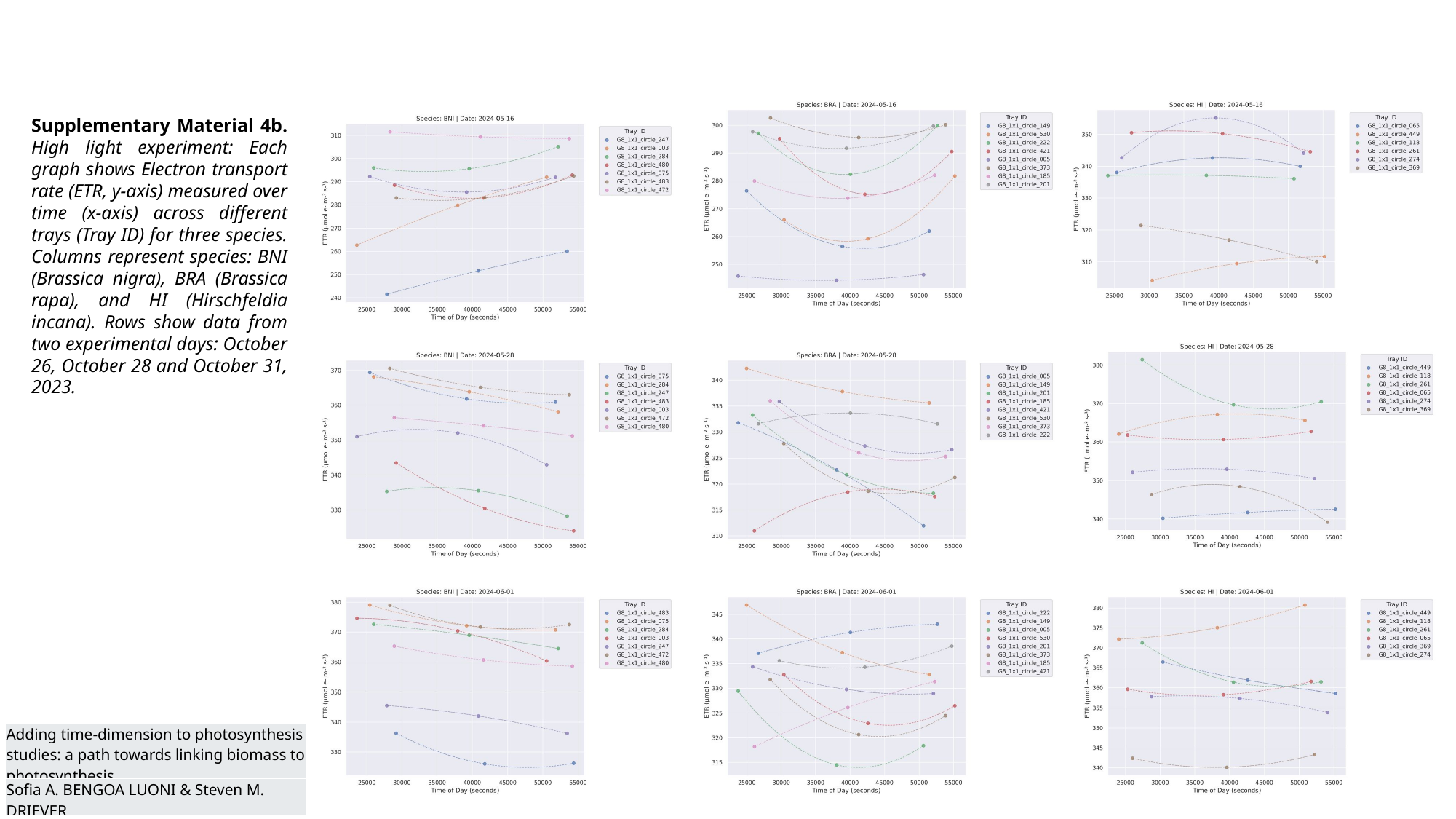

Supplementary Material 4b. High light experiment: Each graph shows Electron transport rate (ETR, y-axis) measured over time (x-axis) across different trays (Tray ID) for three species. Columns represent species: BNI (Brassica nigra), BRA (Brassica rapa), and HI (Hirschfeldia incana). Rows show data from two experimental days: October 26, October 28 and October 31, 2023.
| Adding time-dimension to photosynthesis studies: a path towards linking biomass to photosynthesis |
| --- |
| Sofia A. BENGOA LUONI & Steven M. DRIEVER |
